# Advances in understanding the role of pentraxin-3 in lung infections

**DOI:** 10.3389/fimmu.2025.1575968

**Published:** 2025-04-17

**Authors:** Li Ma, Dongmei Li, Yiyang Wen, Dongmei Shi

**Affiliations:** ^1^ The Laboratory of Medical Mycology, Jining No.1 People’s Hospital, Jining, Shandong, China; ^2^ Department of Microbiology & Immunology, Georgetown University Medical Center, Washington, DC, United States; ^3^ Department of pathology, Jining No.1 People’s Hospital, Jining, Shandong, China; ^4^ Department of Dermatology, Jining No.1 People’s Hospital, Jining, Shandong, China

**Keywords:** pentraxin 3 (PTX3), lung infections, inflammation, complement, biomarkers

## Abstract

Pentraxin-3 (PTX3) is a soluble pattern recognition molecule (PRM) characterized by a C-terminal pentraxin structural domain and a unique N-terminal structural domain. As a key component of the innate immune system, PTX3 can be rapidly released into the extracellular space during microbial invasion and inflammatory responses. It plays a crucial role in regulating complement activation, enhancing the ability of myeloid cells to recognize pathogens, and exerting various immune effects. PTX3 is integral to the regulation of innate immunity, inflammation, and tumor dynamics through its dual function as both a pro-inflammatory and anti-inflammatory mediator depending on the context. This role is closely linked to its diverse molecular and cellular targets. Additionally, PTX3 has been implicated in the pathogenesis of various lung diseases through its involvement in numerous physiological and pathological processes. In this paper, we summarize the complex immunological functions of PTX3 and review the multifaceted roles it plays in the development of infectious lung diseases. Our objective is to highlight the potential for clinical targeting of PTX3 as a biomarker in infectious diseases and to propose it as a viable alternative in future therapeutic strategies.

## 1 Introduction

Lung inflammation caused by various infectious pathogens-including bacteria, fungi, viruses, and atypical organisms-is a common condition affecting the alveoli, distal airways, and interstitium (1). The clinical outcomes of such infections vary widely, depending on the virulence factors of the pathogens and the host’s immune status. These outcomes can range from manageable conditions in otherwise healthy individuals to severe cases with high mortality rates, particularly among critically ill patients or high-risk groups (2). The lungs possess several defense mechanisms to protect the host from pathogens, with innate immunity playing a pivotal role. A key component of this defense involves soluble pattern recognition molecules (PRMs), which are crucial for recognizing and responding to microbial threats (3). However, overactivation of these molecules can lead to tissue damage, exacerbating lung inflammation.

Pentraxin 3 (PTX3), a key component of humoral immunity, is rapidly produced and released by macrophages, dendritic cells (DC), fibroblasts, and activated endothelial cells at sites of infection or inflammation (4, 5). The production PTX3 is regulated by various inflammatory signal molecules, such as IL-1β and TNF-α, as well as Toll-like receptor (TLR) agonists, and microbial components like lipopolysaccharides (LPS), lipoarabinomannan (LAM), and outer membrane proteins (6). These microbial components, which stimulate PTX3 production, are derived from diverse bacteria like *Pseudomonas aeruginosa* (*P. aeruginosa*), *Shigella flexneri* (*S. flexneri*), and urinary tract pathogenic *Escherichia coli* (UPEC); fungi like *Aspergillus fumigatus* (*A. fumigatus*); and viruses like the influenza virus (7).

While PTX3 enhances the body’s defense to clear these pathogens, it also plays an indispensable role in regulating inflammation, tissue remodeling, and anti-tumor responses (8–10). In the context of pulmonary infectious diseases, PTX3 not only promotes the clearance of pathogens by phagocytes but also activates mucosal immune responses against infectious agents. PTX3 thus serves multiple essential functions in respiratory diseases, facilitating both host defense and the regulation of inflammatory processes. In this paper, we summarized the structure, immune role of PTX3, as well as its mechanism in lung infection caused by three main pathogens: bacteria, fungi and viruses. Additionally, we discuss the potential of PTX3 modulators in manage lung infection.

## 2 PTX3 and its immune function

### 2.1 The structure of PTX3

The pentraxin superfamily is characterized by structural domains that are evolutionarily conserved. Based on the length of thei N-terminal regions, pentraxins are categorized into long-chain pentraxins (> 45 kDa) and short-chain pentraxins (25 kDa) (11). Short-chain proteins, including C-reactive protein (CRP) and serum amyloid P component (SAP), are predominately produced by hepatocytes and act as important mediators in regulation of the human immune system (12).

Long-chain pentraxins include Pentraxin 3 (PTX3), Pentraxin 4 (PTX4), neuronal pentraxin 1 (NP1), and neuronal pentraxin 2 (NP2). PTX3 is encoded by three exons, which correspond to a targeting signal peptide, a C-terminal structural domain homologous to classical short pentraxin, and a unique, extended long N-terminal structural pentraxin domain (13, 14). The C-terminal structural domain contains a single N-glycosylation site, crucial for regulating the biological function of the protein in inflammation and innate immunity (15, 16). The N-terminal structural domain contains a convoluted helix and an intrinsically disordered region (17). In addition, the proximal promoter region of the PTX3 gene contains several potential transcription factor binding sites, including specificity protein 1 (Sp1), transcription factor Pu. 1, activator protein-1 (AP-1), nuclear factor of IL-6 (NF-IL-6), and NF-κB (18). The specificity and complexity of PTX3 structure are closely linked to its versatility as a molecule with broad molecular and cellular targets.

### 2.2 Genetic variant of PTX3 and their association with Lung pathogens

**Figure 1:**
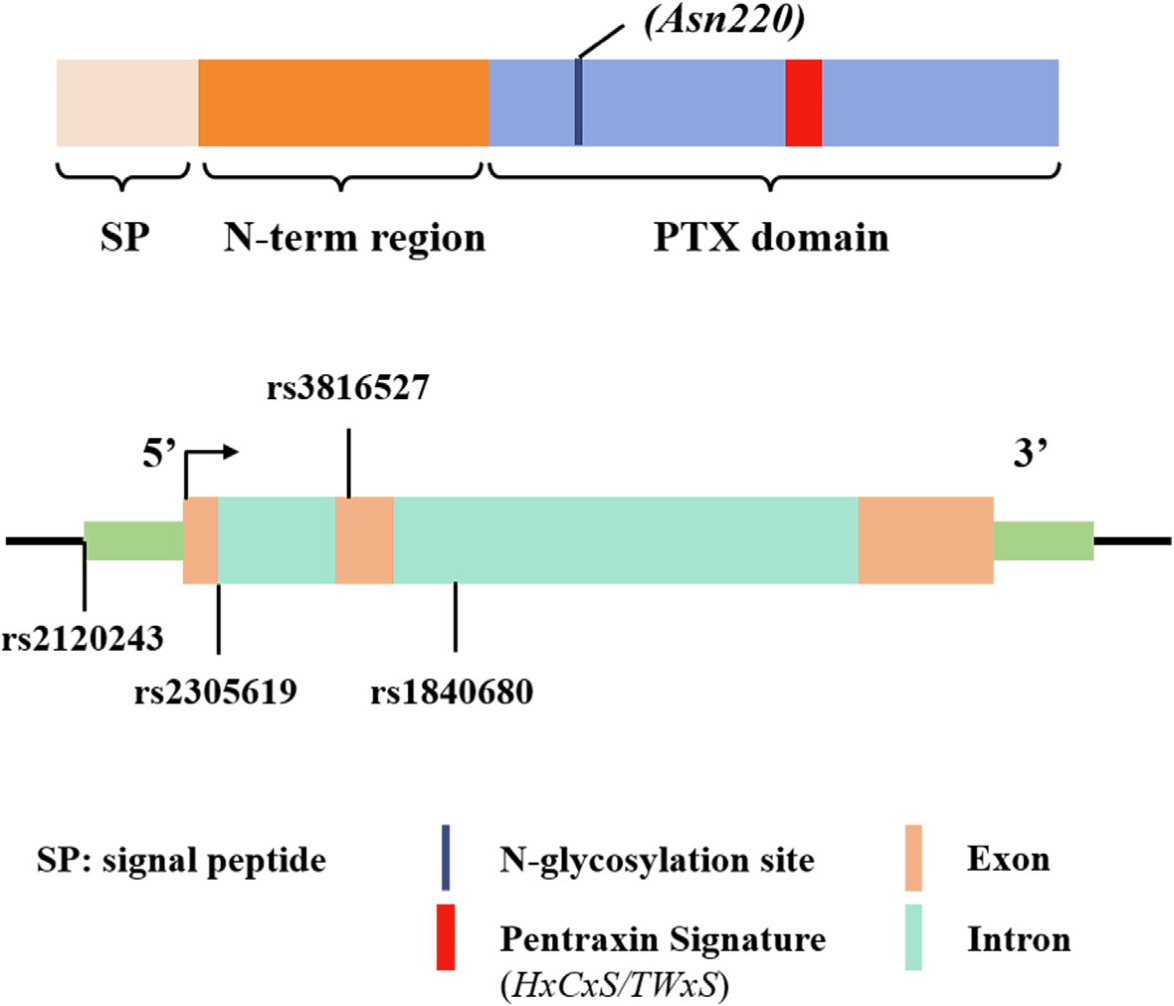
Schematic representation of the gene and protein structure of PTX3, including the approximate locations of the single nucleotide polymorphisms (SNPs) found in various infections. Exons are depicted as solid rectangles, while introns are hollow rectangles.

The PTX3 gene is located in the q25 region of human chromosome 3 and comprises three exons separated by two introns. The first two exons encode the signal peptide and N-terminal domain, and the third exon encodes the C-terminal pentraxin domain, as shown in **Figure 1** (19). Multiple single nucleotide polymorphisms (SNPs) in PTX3 have been identified (20), and these are strongly associated with susceptibility to, and the progression of, infectious and neoplastic diseases (21, 22). Among them, the PTX3 gene variant *rs2120243*, located at a transcription factor binding site, influences transcriptional regulation, thereby affecting circulating plasma PTX3 levels (21, 23).

Previous studies have also shown that two intronic variants of the PTX3 SNPs —*rs2305619* and *rs1840680* —affect PTX3 function (21). The A allele of these variants is linked to elevated plasma PTX3 levels (23) and increased susceptibility to clinical conditions such as infections caused by *P. aeruginosa* and *A. fumigatus* (22, 24, 25). In addition, *rs3816527*is a non-synonymous mutation located on exon 2. This variant affects the function of PTX3 by interfering the N-terminal-mediated PTX3 binding to its ligand (26). These studies emphasize the significance of PTX3 genetic variants in modulating gene expression and protein levels. The detection of PTX3 gene polymorphisms thus may help predict the risk of infection by a variety of pathogens and provide insights on disease susceptibility.

### 2.3 PTX3 in innate and adaptive immune response to infections

PTX3 functions as a soluble pattern recognition receptor and “antibody precursor”, recognizing exogenous microorganisms and modulating the inflammatory responses. - PTX3 binds wide range of ligands, including complement components, growth factors, adhesion molecules (P-selectins), and extracellular matrix glycoproteins, thereby coordinating a interaction between innate immunity and inflammation (27). The complement system, comprising over 30 synergistic soluble proteins, membrane-bound proteins, and complement receptors such as C1q, ficolin, and factor H, is a highly conserved component of the innate immune system. PTX3 exhibits specific binding to these complement components, playing a crucial role in the inflammatory response.

**Figure 2:**
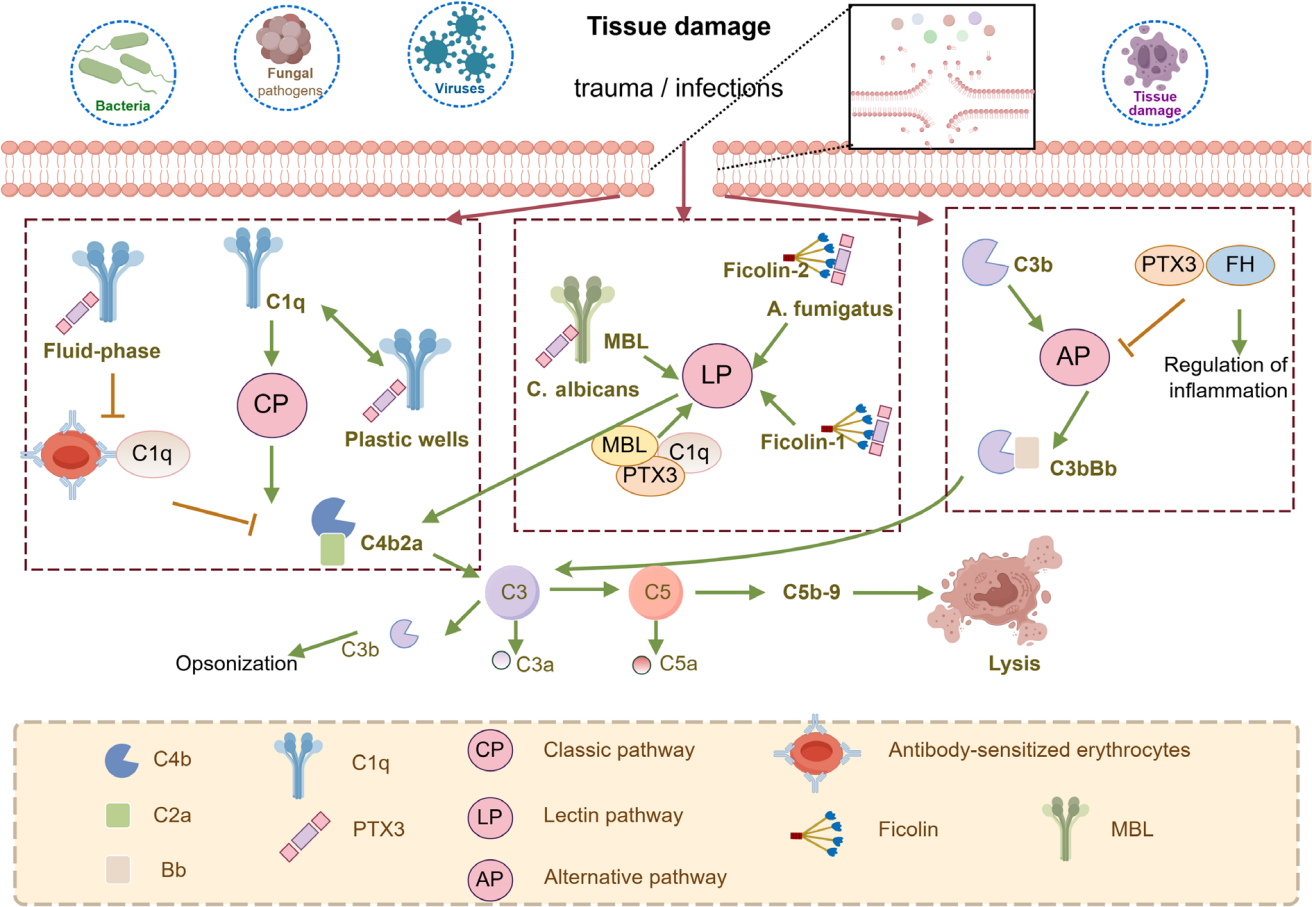
Schematic representation of the involvement of PTX3 in the regulation of complement activity. PTX3 can activate the classical complement pathway (CP) by binding to the C1q globular domain under specific conditions. PTX3 is also involved in the regulation of the lectin pathway (LP) through interactions with MBL and Ficolins. In addition, PTX3 recognizes regulators of complement activation, in particular the most important regulator of the alternative pathway (AP), FH, is involved in the regulation of AP. they trigger the hydrolysis of C3 via C3 convertases (C4b2a for CP and LP, C3bBb for AP) to form C3a, C3b, and C5a, followed by the formation of C5b-9, the membrane attack complex that leads to cell lysis. This figure is drawn by Figdraw.

PTX3 closely interacts with three pathways of complement system, generating distinct context-dependent results (**Figure 2**) (28). In the classical pathway (CP), PTX3 binds to complement component C1q (29) via its globular structural domain. This binding promotes the C3 and C4 deposition, facilitating complement activation on pathogens. Notably, this interaction does not require protein aggregation or the presence of calcium ions (27). However, pre-incubation of PTX3 with C1q in the liquid phase inhibits complement activation by blocking the C1q interaction with immunoglobulins. In the Lectin Pathway (LP), PTX3 directly interacts with ficolin-1, ficolin-2, and mannose-binding lectin (MBL). The Ficolin-1 bound PTX3 promotes the deposition of C4b (30) while the ficolin-2-mediated complement deposition has been observed during *A. fumigatus* infection (31). MBL binds PTX3 and SAP through its collagen-like structural domain, forming an MBL-PTX3 complex that not only recruits C1q, but also enhances C3 and C4 deposition, as well as the phagocytosis of *C. albicans* by polymorphonuclear leukocytes. This MBL- PTX3 complex interacts with both the Lectin and Classical Pathways, amplifying complement activation (32). Furthermore, PTX3 is a unique ligand for Factor H (FH), which recruits FH by recognizing two FH binding sites, SCR7 and SCR19-20. This interaction prevents complement over-activation by downregulating the Alternative Pathway (AP) of complement (29).

In addition to its involvement in complement activation, PTX3 is involved in the recruitment of inflammatory cells. PTX3 has been reported to have significant pro-inflammatory effects, since silencing PTX3 inhibits LPS-induced expression of IL-6, IL-8, IL-1β, and Mucin 5AC (MUCSAC) through suppression of the PI3K/AKT pathway (33). In microglioma cells BV-2, PTX3 knockdown markedly reduces the expression of β-amyloid-induced cytokines TNF, IL-1β, IL-6, iNOS, and CD86 and COX-2 (34).

Interestingly, PTX3 also exerts anti-inflammatory effects under certain conditions. In a model of LPS-induced acute lung injury, PTX3 deficiency significantly exacerbates the lung injury, accompanied by increased neutrophil infiltration and elevated plasma levels of TNF-α and monocyte chemotactic protein-1 (MCP-1) (35). Similarly, exogenous PTX3 stimulation in human PBMCs increased the production of the anti-inflammatory cytokine IL-10, without affecting production of pro-inflammatory cytokines IL-1β, IL-6, and TNF-α (36). Beyond its direct induction of inflammatory cytokines, PTX3 also modulates inflammation-related signaling pathways. In *A. fumigatus* infection, PTX3 binds to myeloid differentiation protein 2 (MD-2) *in vitro* and exerts its protective antifungal activity *in vivo* by activating the anti-inflammatory TLR4/TRIF signaling pathway (37).

This dual immune effects of PTX3 suggests its critical function in regulating antimicrobial activity and controlling immunopathogenesis.

## 3 Role of PTX-3 in bacterial infections of the lungs

PTX3 plays a protective role in bacterial lung infections through multiple mechanisms, including enhancing pathogen recognition, promoting phagocytosis, and modulating inflammatory responses. Additionally, PTX3 serves as a valuable biomarker for assessing disease severity and prognosis. Below we highlight a few typical bacterial infection examples.

### 3.1 Pseudomonas aeruginosa

*Pseudomonas aeruginosa* is a common lung colonizer that can cause acute and chronic respiratory infections, particularly in patients with underlying conditions such as cystic fibrosis (CF). Several studies have demonstrated that *P. aeruginosa* infection significantly upregulates PTX3 expression (25, 38–40). Notably, serum PTX3 levels are significantly higher in CF patients compared to healthy individuals (38). Furthermore, an animal study has confirmed that PTX3-deficient mice exhibit increased susceptible to infection with laboratory strains of *P. aeruginosa*.

Mechanistically, PTX3 protects against *P. aeruginosa* infections by modulating inflammatory responses and enhancing pathogen clearance. Moalli F et al.demonstrated that PTX3 reduces lung infections in mice by decreasing levels of pro-inflammatory cytokines (e.g.,IL-1β) and chemokines (e.g., CXCL1, CXCL2, and CCL2), as well as by reducing airway neutrophil recruitment in a mouse model infected with the clinical strain RP73, isolated from CF patients (41). PTX3 enhances regulatory phagocytosis in a complement- and Fc gamma receptor (FCGR)-dependent manner. Genetic variations in PTX3 also play a role in *P. aeruginosa* colonization. Chiarini M et al. (25)found significant differences in the frequency of PTX3 haplotypes among 172 Caucasian CF patients, suggesting that genetic variants in PTX3 influence the lung colonization of *P. aeruginosa* in CF patients.

Heat shock proteins (HSP)70 chaperone proteins, conserved across prokaryotic and eukaryotic cells, induced under various stresses and infections, have been implicated in PTX3 regulation (42). DnaK,a homologue of HSP70, was shown to stimulate PTX3 expression through the NF-κB and extracellular signal-regulated kinase (ERK) signaling pathways. Notably, ERK activation is negatively regulated by NF-κB, indicating crosstalk between these pathways. The same study suggests that *P. aeruginosa* DnaK acts as a pathogen-associated molecular pattern that enhances host defense responses by promoting PTX3 production (43).

### 3.2 Klebsiella pneumoniae

Nosocomial *Klebsiella pneumoniae* (*K. pneumoniae*) is a leading cause of community-acquired pneumonia, particularly in individuals with impaired lung defenses and functions (44, 45). Similar to other bacterial infections, PTX3 levels have been found to correlate with the progression of *K. pneumoniae* infection. Using a PTX3 transgenic mouse model, Soares et al. (46) demonstrated that PTX3 exerts dual effects during lung infection. In mice receiving low-dose bacterial inoculation, PTX3 overexpression led to increased TNF-α expression and neutrophil infiltration, which significantly reduced bacterial loads and enhanced resistance to respiratory infections. Conversely, in mice challenging with high-dose inoculations, PTX3 overexpression was associated with increased lethality. This highlights that the effects of PTX3 are dose-dependent and influenced by the severity of the inflammatory response, suggesting a fine balance between protective and detrimental outcomes in *K. pneumoniae* infections.

Using PTX3-deficient mice, PTX3’s role in innate immunity against *K. pneumoniae* was investigated in a recent study. Consistent with previous findings, local and systemic PTX3 expression was induced following lung infection, accompanied by increased TNF-α and IL-1β levels. However, unlike in *P. aeruginosa* infections, PTX3 did not directly interact with *K. pneumoniae* or promote conditioned phagocytosis. This discrepancy may be contributed to the pathogen’s escape mechanisms, such as polysaccharide capsule of *K. pneumoniae* (47).

Analysis of the susceptibility of wild-type, PTX3-deficient, complement C3-deficient and Ptx3^-/-^/C3^-/-^ mice to infection revealed that PTX3 exerts its protective effects against *K. pneumoniae* in a complement-independent manner. Histopathological analysis of the infected lungs showed increased fibrin deposition and greater consumption of circulating fibrinogen in the lungs. These findings suggest that PTX3 controls *K. pneumoniae* infection primarily by modulating the inflammatory response and tissue damage (48).

### 3.3 Streptococcus pneumoniae

*Streptococcus pneumoniae*(*S. pneumoniae*)is a gram-positive diplococcus and a major pathogen responsible for approximately 2 million deaths annually worldwide (49). Toxigenic strains of *S. pneumoniae* secrete pneumolysin, a toxin that upregulates PTX3 expression in mouse epithelial cells and lungs via the JNK1/2 MAPK pathway. This interaction enhances the inflammatory response and induces the expression of inflammatory cytokines in the lungs (50).

PTX3 expression is significantly elevated in patients with bacterial infections, including chronic obstructive pulmonary disease and community-acquired pneumonia (51, 52). Notably, PTX3 levels in pleural fluid are valuable for identifying parapneumonic effusion (53). Furthermore, studies measuring PTX3 levels in the plasma of patients with bacterial infections and aseptic meningoencephalitis revealed significantly higher PTX3 levels in those with bacterial infection (54). These findings suggest that PTX3 has a potential as a biomarker for *S. pneumoniae* infections, providing valuable support for clinical diagnosis. However, further studies are needed to clarify the molecular mechanisms underlying PTX3-mediated inflammatory regulation during *S. pneumoniae* infections.

### 3.4 M. tuberculosis

A study investigating 70 biomarkers associated with infection and inflammation in tuberculosis patients identified PTX3 as one of seven elevated proteins linked to disease severity, smear grading, and extensive imaging lesions (55). Similarly, a separate study involving 220 tuberculosis patients demonstrated significantly higher plasma PTX3 levels in tuberculosis patients compared to healthy controls (56). Notably, PTX3 levels decreased significantly in 186 patients who were cured by the end of treatment, whereas levels rose again in all 34 patients who experienced treatment failure. Although PTX3 is not specific for diagnosing tuberculosis, its detection can help monitor disease activity and treatment efficacy.

PTX3 gene polymorphisms have also been associated with increased susceptibility to *M. tuberculosis* infections *(* 57). In a study of 213 tuberculosis patients in West Africa, two SNPs—rs1840680 and rs2305619 (**Figure 1**) —in the PTX3 gene were found to be less frequent in the TB group compared to healthy controls, indicating that these genotypes may confer protection against tuberculosis infection (58). Additionally, exposure to the Bacillus Calmette-Guérin (BCG) vaccine was associated with increased PTX3 levels in human lung disease specimens (59), suggesting that PTX3 might play a role not only as a marker of disease state but also as a potential protective mechanism, though this mechanism remains to be fully understood.

Collectively, these studies advance our understanding of immune response due to bacterial infections and highlights PTX3 as a potential target for therapeutic strategies to control bacterial infections.

## 4 Role of PTX3 in fungal infections of the lungs

**Figure 3:**
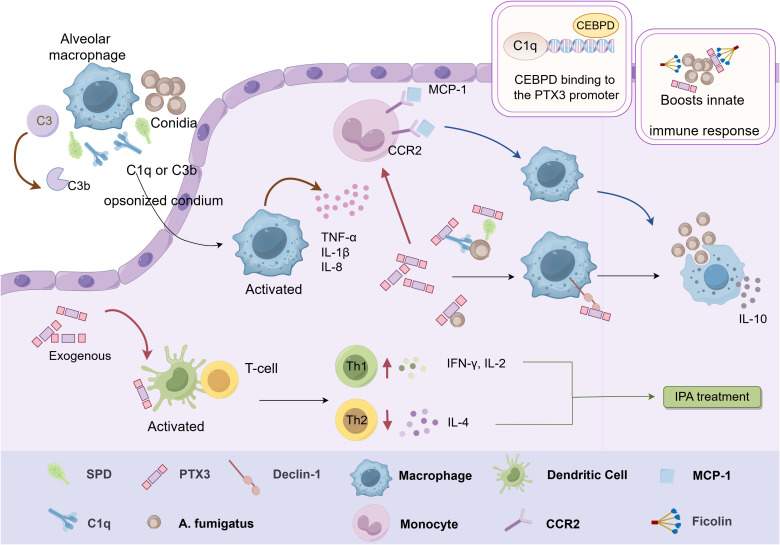
Schematic representation of the immunomodulatory function of PTX3 in *Aspergillus*. Inhaled conidia conditioned by SP-D, C1q or C3b are phagocytosed by alveolar macrophages, which stimulate the release of various cytokines from macrophages. PTX3 induces mononuclear phagocytes to release the chemokine MCP-1 in conjunction with CCR2, which potentiates the macrophage antifungal response. Exogenous PTX3 activates dendritic cells and induces an imbalance in the Th1/Th2 response, resulting in effective mitigation of IPA. PTX3 also interacts with ficolin on the surface of conidia and enhances the activation of LPs, which promotes innate immunity against *A. fumigatus*. In addition, CEBPD can activate PTX3 by binding to the PTX3 promoter region, thereby enhancing PTX3 expression and promoting phagocytosis by macrophages. This figure is drawn by Figdraw.

PTX3 plays a crucial role in immune defense, particularly innate immunity, against fungal infections in the lungs by enhancing macrophages phagocytosis, promoting complement deposition, and forming protein complexes to facilitate fungal clearance (**Figure 3**).

Invasive pulmonary aspergillosis (IPA) is a severe condition characterized by the dissemination of fungal hyphae in lung and surrounding tissues, with high morbidity and mortality rates (60–62). The host’s intrinsic immune system is critical for clearing inhaled *Aspergillus fumigatus* spores, primarily through immune cells such as macrophages, neutrophils, and dendritic cells (63). Studies have shown that PTX3-deficient mice exposed to *A. fumigatus* are highly susceptible to IPA, with all mice succumbing within three days. These PTX3-deficient mice displayed significantly elevated fungal loads and severe inflammatory injury in the lung. Notably, the administration of recombinant PTX3 could reverse these outcomes. Mechanistic studies revealed that PTX3 selectively recognizes and binds galactomannan (GM), a polysaccharide in *A. fumigatus* cell wall. This binding activates the complement system and promotes macrophage phagocytosis, thereby enhancing resistance to *A. fumigatus* infection (64).

Susceptibility to IPA is also associated with an imbalance in Th1/Th2 response. PTX3-deficient mice exhibited increased levels of Th2-type cytokine IL-4 and decreased levels of Th1-type cytokine IL-2 and IFN-γ. Administration of exogenous PTX3 in PTX3-deficient mice activated DCs and induced Th1 responses, effectively mitigating IPA (64). PTX3 further enhances phagocytosis by interacting with dendritic cell-associated C-type lectin-1 (Dectin-1), amplifying the immune response against *A. fumigatus*. Notably, blocking the Dectin-1 receptor impairs phagocytosis in PTX3 transgenic mice (65). In addition, PTX3 induces mononuclear phagocytes to release chemokine MCP-1/CCL-2, which recruits monocytes to lung tissues, enhancing antifungal responses (66).

PTX3 mitigates harmful inflammation by recognizing *A. fumigatus* directly or indirectly through interactions with other humoral PRMs. Among these PRMs, fibronectin (ficolin) glycoproteins play a role by binding oligosaccharides on pathogenic microorganisms and activating the complement lectin pathway (LP) (67). However, both ficolin-1 binding complex and LP activation with PTX3 were not observed during exposure to *A. fumigatus* conidia (68). Instead, PTX3 interacts with ficolin-2 on the conidial surface, enhancing LP activation and contributing to innate immunity against *A. fumigatus (* 69). Although the interaction between PTX3 and ficolin-3 has been identified, its functional implications in antifungal immunity remain unclear (31). A recent study confirmed that PTX3 recognizes germinating conidia via galactosaminogalactan, a polysaccharide on the *A. fumigatus* cell wall. For dormant conidia, other humoral PRMs such as Surfactant Associated Protein D (SP-D) and complement proteins C1q and C3b facilitate PTX3 binding. Interestingly, PTX3 binding to *A. fumigatus* conidia significantly decreased pro-inflammatory cytokine/chemokine production and increased IL-10 release (70). This finding suggests a nuanced role of PTX3 in modulating inflammation by balancing immune defense with the prevention of excessive inflammatory damage.

Furthermore, PTX3 gene polymorphisms significantly influence PTX3 expression levels and the risk of IPA (71–75). Recent studies have shown that specific polymorphisms in PTX3 promoter may affect its binding with transcriptional enhancer-binding proteins (76), such as CCAAT/enhancer-binding proteins (CEBPs), influencing PTX3 transcription efficiency. It has been shown that CEBPD activates PTX3 by binding to its promoter region following *A. fumigatus* infection, thereby enhancing PTX3 expression and facilitating macrophage phagocytosis (77).

IPA mainly affects immunocompromised patients, including solid organ transplant recipients and those undergoing hematopoietic stem cell transplantation (78). In bone marrow transplantation patients, PTX3 promotes the accumulation of myeloid and lymphoid cells in the lung tissue, accelerating the immune function recovery. The efficacy of antifungal treatment for pulmonary aspergillosis is enhanced when PTX3 is administered alongside antifungal drugs (79). Additionally, PTX3 gene polymorphisms are associated with the incidence of IPA in hematopoietic stem cell transplant recipients, potentially due to defects in neutrophil antifungal activity.

Recent reports indicate a rising incidence of IPA in patients with chronic obstructive pulmonary disease (COPD), highlighting COPD as a significant risk factor for IPA (22, 80). Studies on PTX3 gene polymorphisms and susceptibility to *Aspergillus* in patients with COPD have revealed that the frequency of the rs1840680 AA genotype is significantly higher in those with comorbid IPA compared to COPD patients without IPA. Additionally, peripheral blood PTX3 levels are lower in COPD patients with the AA phenotype at the rs1840680 locus, suggesting an increased risk of IPA in this population (81). These findings support that potential of plasma PTX3 levels as a promising diagnostic indicator for IPA in COPD patients.

Although the results of the available studies confirmed the potential of PTX3 as a biomarker for early diagnosis of IPA in non-neutropenic patients and COPD combined with *A. fumigatus* infection,. studies on the correlation between PTX3 and the prognosis of IPA are still limited. The prognostic value of PTX3 in pulmonary aspergillosis needs to be further explored.

## 5 The role of PTX3 in viral infections

Studies also confirmed that PTX3 plays a vital role in the immune response to variable viruses, including SARS-CoV-2, H3N2 influenza, and human cytomegalovirus, through activating complement cascades and promoting pathogen recognition by myeloid cells.

### 5.1 The role of PTX3 in SARS-CoV-2 lung infection

SARS-CoV-2, the virus responsible for the COVID-19 pandemic, elicits a robust immune response of the innate immune system (82, 83). Several studies have shown PTX3 levels as a biomarker for predicting disease severity and mortality risk in COVID-19 patients (57, 84, 85). Immunohistochemical analysis of autopsy samples from COVID-19 lungs revealed high PTX3 expression in bone marrow monocytes and endothelial cells. Plasma PTX3 level was even identified as a significant independent predictor of 28-day mortality in hospitalized patients, demonstrating superior over traditional inflammatory markers (85).

A prospective cohort study also confirmed that plasma PTX3 effectively predicts the risk of respiratory failure and death within 30 days for COVID-19 patients, regardless of treatment with remdesivir or dexamethasone (86). Additionally, PTX3 has been identified as a low-cost/low-tech early biomarker for detecting secondary bacterial or fungal infections in COVID-19 patients (87), aiding in identification of individuals at high risk for community- or hospital-acquired infections and guiding an early antimicrobial therapy.

Mechanistically, PTX3 binds specifically to the nucleocapsid protein (NP) of SARS-CoV-2 through its N-terminal structural domain in a dose-dependent manner. However, it does not exhibit direct anti-SARS-CoV-2 activity. Whether PTX3 participates in nucleocapsid-mediated complement activation and cytokine production remains to be elucidated (81). SNP analysis revealed that individuals with the rs1840680 (1449A/G) AG genotype (**Figure 1**) have greater protection against severe COVID-19 compared to those with the AA genotype. Additionally, PTX3 level was positively correlated with the expression of inflammatory cytokines such as IL-6, IL-8, and IL-10, suggesting a role of PTX3 in modulating the complement system and inflammatory cytokines (88). Differences in PTX3 expression were observed between deteriorating and recovering COVID-19 patients, with associated changes in the JAK-STAT, NF-κB, and MAPK signaling pathways, all contributing to inflammatory responses (89). These findings demonstrate the critical role of PTX3 in the immunopathology of COVID-19, however, the specific mechanism of action remains unclear and further studies are needed.

### 5.2 The role of PTX3 in influenza lung infection

Respiratory influenza viruses are one of the most common human pathogens and are responsible for seasonal morbidity and mortality worldwide. PTX3, a soluble pattern-recognition receptor, exhibits potent antiviral activity against influenza virus both *in vivo* and *ex vivo*, mediating a range of antiviral responses. These responses include inhibition of hemagglutination, neutralization of the virus and inhibition of viral neuraminidase activity. Studies have demonstrated that PTX3 is rapidly upregulated following H3N2 influenza infection. It binds to viral hemagglutinin and prevents viral attachment to host cells, thereby inhibiting viral replication in the lung. Animal models further confirm PTX3’s importance since PTX3 knockout mice are more susceptible to the virus (90).

PTX3 also participates in neutralization of influenza A viruses (IAV). The anti-IAV activity is achieved by increasing salivary acids that can neutralize the hemagglutinin (HA) glycoprotein (91). Studies have revealed that the specificity of HA and viral neuraminidase for particular sialic acid linkages influences the susceptibility of H1N1, H3N2, and H7N9 strains to PTX3’s antiviral effects, suggesting a variable effectiveness of PTX3 to different IAV subtypes (92).

### 5.3 The role of PTX3 in human cytomegalovirus lung infection and other viruses

Human cytomegalovirus (HCMV), a member of the Herpesviridae family, is a ubiquitous opportunistic pathogen capable of entering a latent phase after the clearance of primary infection. It often reactivates when the body’s immunity is suppressed (93–95). PTX3 inhibits the activity of HCMV virus upon binding to hemagglutinin on viral surface and reduces the invasiveness and infectivity of the virus *in vitro*. This mechanism appears to be mediated by TLR9/MyD88-independent viral recognition that activates interferon regulatory factor 3 (IRF3) in DCs and promotes IL-12/IFN-dependent immune pathways. In CMV-infected mice, exogenous PTX3 treatment significant reduced lung viral titers and virus-induced inflammatory cell infiltration while mitigating lung parenchymal destruction (96).

Clinical studies have also highlighted the role of PTX3 gene polymorphisms in the context of HCMV infection. For instance, patients receiving allogeneic transplants, such as stem cell and kidney transplants (97, 98), especially exhibit an increased risk of CMV reactivation if their donors carry PTX3 haplotypes such as h2/h2 (rs2305619, rs3816527) (**Figure 1**). Genetic variation in donor PTX3 in patients undergoing hematopoietic stem cell transplantation thus has the prognostic value to predicate the risk of CMV reactivation (97).

Using PTX3 knockout mice infected with Mouse Hepatitis Virus-1 (MHV-1) has demonstrates that PTX3 is essential for modulating recruitment of neutrophils and macrophages to the lungs. In its absence, this recruitment significantly aggravates lung injury. The elevation of inflammatory factors such as IL-6, MCP-1 and Macrophage Inflammatory Protein 1Beta (MIP) were observed in knockout mice. In contrast, treatment with PTX3 effectively reversed these inflammatory symptoms, reducing cytokine levels and ameliorating airway injury (99). In addition, significant PTX3 expression has also been observed in infections caused by hepatitis B virus, hepatitis C virus and dengue virus infections (100–102).

In conclusion, PTX3 responds rapidly to viral invasion, exerting antiviral effects by regulating complement activation and enhancing pathogen recognition by myeloid cells. Following viral infection, PTX3 concentrations increase quickly and correlate closely with disease severity, suggesting it as a potential biomarker for the diagnosis and prognosis of viral infections in the lungs. In addition, PTX3 may serve a therapeutic role by binding to viruses to reduce intracellular viral load and activating relevant immune pathways.

## 6 Conclusion and perspective

PTX3 has recently garnered significant attention due to its multiple functions in innate immunity. As a soluble pattern recognition receptor and acute phase response protein, PTX3 serves as a crucial component of immune response against infectious pathogens, showing promise as a novel biomarker for diagnosing various lung diseases, assessing severity, and predicting prognosis. Unlike traditional markers like CRP, PTX3 occurs earlier at the site of inflammation and injury.

PTX3 recognizes and binds to various pathogens, including bacteria, fungi, and viruses, thereby promoting phagocytosis by macrophages and other immune cells. It enhances complement activation, regulates inflammatory responses, amplifies immune signaling, activates adaptive immunity, and aids in pathogen clearance. These characteristics position as a potential therapeutic target for various infectious and inflammatory diseases. However, the dual anti-inflammatory and pro-inflammatory roles of PTX3 present a complex dynamic require further investigation.

In conclusion, while PTX3 holds great promise as a biomarker and therapeutic target, more in-depth studies are required to fully unlock its clinical value and address its complex roles in infectious and inflammatory diseases. Key studies should focus on establishing its reliability, optimizing its clinical applications, and exploring strategies to harness its therapeutic potential.
